# Efficacy of microbicides for inactivation of Ebola–Makona virus on a non-porous surface: a targeted hygiene intervention for reducing virus spread

**DOI:** 10.1038/s41598-020-71736-x

**Published:** 2020-09-17

**Authors:** Todd A. Cutts, Catherine Robertson, Steven S. Theriault, Raymond W. Nims, Samantha B. Kasloff, Joseph R. Rubino, M. Khalid Ijaz

**Affiliations:** 1 Applied Biosafety Research Program, Canadian Science Centre for Human and Animal Health, 1015 Arlington Street, Winnipeg, MB R3E 3P6 Canada; 2 J. C. Wilt Infectious Diseases Research Centre, Public Health Agency of Canada, 745 Logan Street, Winnipeg, MB R3E 3L5 Canada; 3 Department of Microbiology, The University of Manitoba, Winnipeg, MB R3T 2N2 Canada; 4 RMC Pharmaceutical Solutions, Inc., 1581 Lefthand Circle, Suite A, Longmont, CO 80501 USA; 5 Global Research and Development for Lysol and Dettol, Reckitt Benckiser LLC, One Philips Parkway, Montvale, NJ 07645 USA; 6 Medgar Evers College of the City University of New York (CUNY), 1650 Bedford Ave, Brooklyn, NY 11225 USA

**Keywords:** Microbiology, Diseases, Health care

## Abstract

Microbicides play critical roles in infection prevention and control of Ebola virus by decontaminating high-touch environmental surfaces (HITES), interrupting the virus-HITES-hands nexus. We evaluated the efficacy of formulations containing different microbicidal actives for inactivating Ebola virus–Makona strain (EBOV/Mak) on stainless-steel carriers per ASTM E2197-11. Formulations of sodium hypochlorite (NaOCl) (0.05–1%), ethanol (70%), chloroxylenol (PCMX) (0.12–0.48% by weight) in hard water, and a ready-to-use disinfectant spray with 58% ethanol (EDS), were tested at contact times of 0, or 0.5 to 10 min at ambient temperature. EBOV/Mak was inactivated (> 6 log_10_) by 70% ethanol after contact times ≥ 2.5 min, by 0.5% and 1% NaOCl or EDS (> 4 log_10_) at contact times ≥ 5 min, and by 0.12–0.48% PCMX (> 4.2 log_10_) at contact times ≥ 5 min. Residual infectious virus in neutralized samples was assessed by passage on cells and evaluation for viral cytopathic effect. No infectious virus was detected in cells inoculated with EBOV/Mak exposed to NaOCl (0.5% or 1%), PCMX (0.12% to 0.48%), or EDS for ≥ 5 min. These results demonstrate ≥ 6 log_10_ inactivation of EBOV/Mak dried on prototypic surfaces by EDS or formulations of NaOCl (≥ 0.5%), PCMX (≥ 0.12%), or 70% ethanol at contact times ≥ 5 min.

## Introduction

The Ebola virus has continued to re-emerge in lethal outbreaks, with the most recent occurring in the Democratic Republic of the Congo, Africa in May 2018^1^. This most recent outbreak, and the outbreaks occurring between 2014 and 2016, emphasize the need^2^ for effective approaches for reducing the spread of the disease from community to community and from nation to nation. Ebola virus disease is listed in the World Health Organization’s List of Blueprint Priority Diseases, as “… given their potential to cause a public health emergency and the absence of efficacious drugs and/or vaccines, there is an urgent need for accelerated research and development…”^3^.

The Ebola virus may be transmitted, in part, by contact with environmental surfaces (fomites) contaminated with secretions and excretions from infected individuals^4^. For instance, objects in the vicinity of infected patients (including IV insertion site, patient’s skin, mattress, clothes, blanket, digestive losses bucket, IV drip stand, floor, and healthcare workers’ personal protective equipment) have been shown to be contaminated with Ebola virus RNA^5^. Considering this, an important intervention for limiting viral dissemination may involve the use of an effective virucidal agent for disinfecting surfaces contaminated with Ebola virus, thereby mitigating the risk of transmission of the virus to healthy individuals, including health-care workers.

The Ebola virus is a member of the *Filoviridae* family, and is an enveloped virus. As such, the Ebola virus should be relatively susceptible to a variety of microbicidal inactivation approaches^6^. In view of the lethality of the virus, the United States Centers for Disease Control and Prevention (CDC) offers the following guidance^7^: “…selection of a disinfection product with a higher potency than what is normally required for an enveloped virus is being recommended at this time. EPA-registered hospital disinfectants with label claims against non-enveloped viruses (noroviruses, rotavirus, adenovirus, poliovirus) are broadly antiviral and capable of inactivating both enveloped and non-enveloped viruses.” The United States Environmental Protection Agency (EPA) requires that claims for efficacy of a product for an emerging enveloped virus include that the product be approved for inactivating at least one large or one small non-enveloped virus^8^.

The virucidal efficacy of microbicides for Ebola virus is usually determined in studies involving virus suspended in a liquid matrix. In addition, many studies have made use of surrogate viruses such as bacteriophages, enveloped viruses (animal coronaviruses, influenza viruses), or non-enveloped viruses such as caliciviruses or picornaviruses. As a result, there are few reports of the efficacy of inactivation of Ebola virus dried onto carriers (i.e., prototype environmental surfaces). The testing of virucidal efficacy for Ebola virus surrogates theoretically should ensure efficacy for inactivation of the Ebola virus. Despite this, testing conducted specifically with Ebola virus itself is needed to confirm the results obtained with surrogate viruses. In this study, virucidal investigations were performed using fully pathogenic Ebola virus at the Canadian Science Centre for Human and Animal Health Biosafety Level 4 (BSL-4) facility.

**Figure 1:**
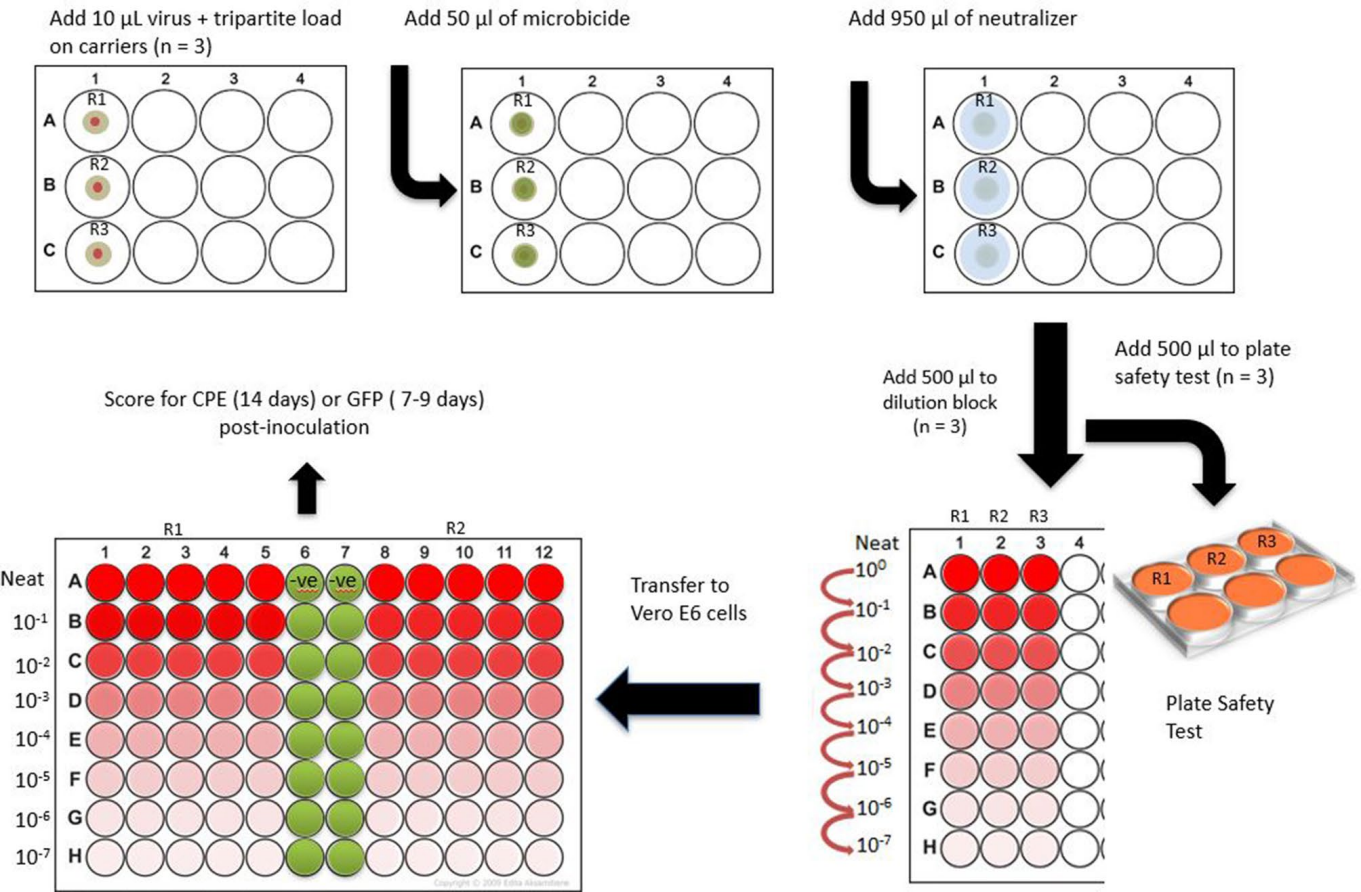
Schematic representation of the carrier inactivation efficacy testing methodology employed.

In this paper, we have conducted studies on the inactivation of Ebola virus–Makona variant (EBOV/Mak), dried onto steel carriers in the presence of an organic load, per American Society for Testing and Materials (ASTM) method ASTM E2197-11^9^ (Fig. 1). Organic soil loads^9,10^ are added to the study design in order to better model inactivation by microbicides of pathogens dried on relevant matrices such as human sputum or blood. Use of hard water as diluent was included in the study design to simulate water hardness in the field. We have compared the efficacy data recently collected for para-chloro-meta-xylenol (PCMX) and a formulated disinfectant spray containing 58% ethanol (EDS) to data published previously^11^, at the same testing facility and using the same methodology, for 70% ethanol and for varying concentrations of sodium hypochlorite (NaOCl). We have reported both sets of results within this article in order to facilitate direct comparisons of virucidal efficacy for the various microbicidal actives.

In addition to the methodology described in the ASTM standard, we also evaluated any residual infectious virus following exposure to microbicides through inoculation of undiluted neutralized test sample into cultures of Vero E6 indicator cells. This was done to evaluate the possibility of virus being present at levels lower than the limit of detection of the tissue culture infectious dose_50_ (TCID_50_) assay performed in Vero E6 cells per the ASTM standard^9^.

## Results

### Neutralization effectiveness evaluation

During the evaluation of possible neutralizing agents, it was determined that PCMX could be neutralized with Letheen broth, and the disinfectant alcohol spray could be neutralized with DMEM + 10% FCS + 10 units/mL penicillin/streptomycin (see Supplemental Materials). It was previously shown that 70% ethanol could be neutralized with Dulbecco’s minimum essential medium (DMEM)^11^, and that NaOCl could be neutralized by 1% sodium thiosulfate^11^.

### Efficacy of inactivation of EBOV/Mak on carriers by PCMX

**Figure 2:**
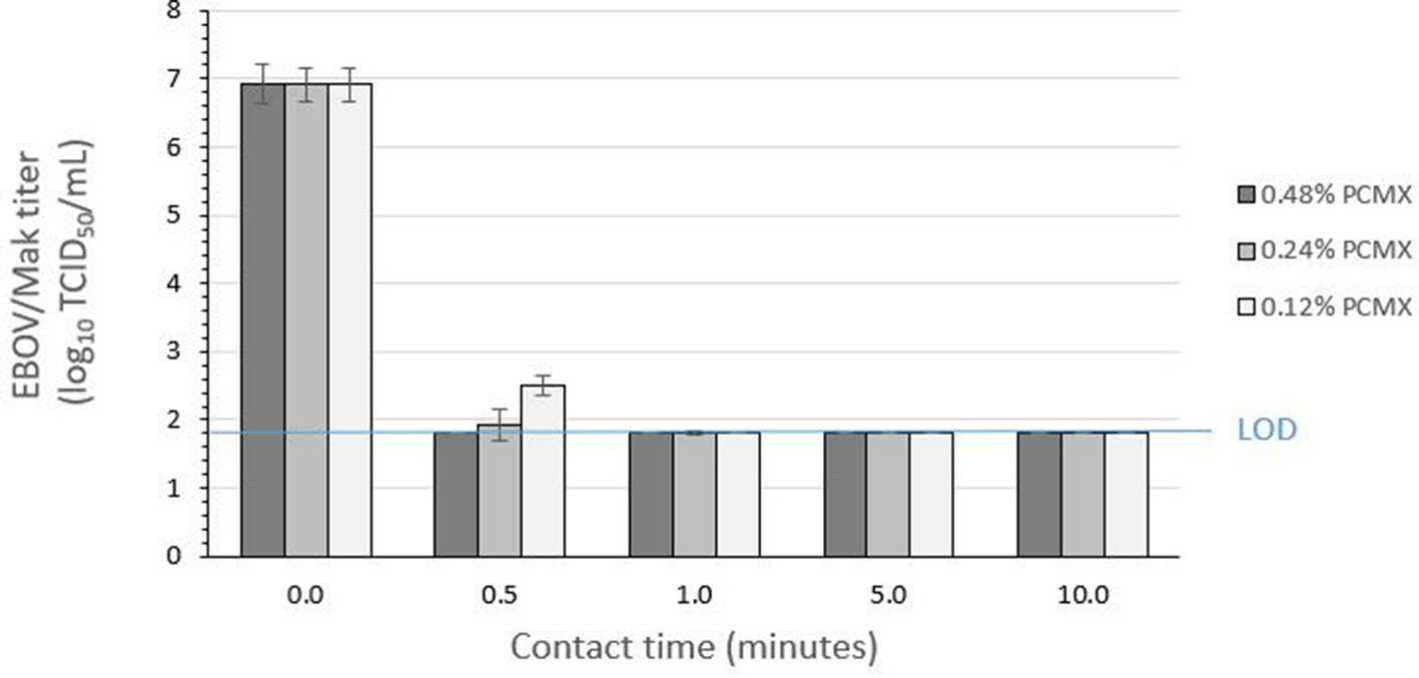
Time kinetics for EBOV/Mak virus inactivation by varying concentrations of PCMX at ambient temperature. The values indicated represent the mean ± standard deviation log_10_ titer of the post-neutralization samples determined in Vero E6 cells (n = 3 technical replicates obtained for 3 lots of PCMX). The input virus titer in tripartite soil load was found to be 6.9 ± 0.3 log_10_ TCID_50_/mL. The limit of detection (LOD) of the assay (1.8 log_10_ TCID_50_/mL) was determined by cytotoxic effects of the undiluted neutralized samples on Vero E6 cells.

The efficacy of PCMX for inactivating EBOV/Mak dried within an organic load on stainless steel carriers was evaluated per ASTM E2197-11^9^. Three lots of PCMX were evaluated at three concentrations each (0.12%, 0.24%, and 0.48%) in hard water, with contact times of 0.5, 1, 5, and 10 min at ambient temperature (~ 21 °C). An initial virus load of ~ 6.9 log_10_ tissue culture infectious dose_50_ (TCID_50_) was dried on the carriers and then exposed to PCMX. At each concentration evaluated, the infectious EBOV/Mak titer recovered from the carriers was reduced by > 5 log_10_ to the LOD of the assay within five min (Fig. 2).

The results of the plate safety test for PCMX are shown in Table 1. Detectable infectious EBOV/Mak was still present after contact times of 0.5 and 1 min with the lowest two PCMX concentrations. Inactivation was complete (no virus detected) for all PCMX concentrations within 5 min contact time on carriers (Fig. 2 and Table 1).

**Table 1:** Plate safety test results for inactivation of EBOV/Mak by PCMX concentrations.

Test condition (contact time)	0.48% PCMX			0.24% PCMX			0.12% PCMX		
	Lot 1	Lot 2	Lot 3	Lot 1	Lot 2	Lot 3	Lot 1	Lot 2	Lot 3
Negative control	−, −, −	−, −, −	−, −, −	−, −, −	−, −, −	−, −, −	−, −, −	−, −, −	−, −, −
N + PCMX	−, −, −	−, −, −	−, −, −	−, −, −	−, −, −	−, −, −	−, −, −	−, −, −	−, −, −
PCMX (0.5 min)	+, +, +	+, +, +	+, +, +	+, +, +	+, +, +	+, +, +	+, +, +	+, +, +	+ , + , +
PCMX (1 min)	+, +, +	+, +, +	+, +, +	+, +, +	+, +, +	+, +, +	+, +, +	+, +, +	+ , + , +
PCMX (5 min)	−, −, −	−, −, −	−, −, −	−, −, −	−, −, −	−, −, −	+, −, −	−, −, −	−, −, −
PCMX (10 min)	−, −, −	−, −, −	−, −, −	−, −, −	−, −, −	−, −, −	−, −, −	−, −, −	−, −, −

The Vero E6 cultures were inoculated with undiluted neutralized samples and passaged twice. +, viral GFP observed; −, viral GFP not observed, or no cytotoxicity observed for N + PCMX (neutralizer + PCMX cytotoxicity control).

### Efficacy of inactivation of EBOV/Mak on carriers by ethanol disinfectant spray

**Figure 3:**
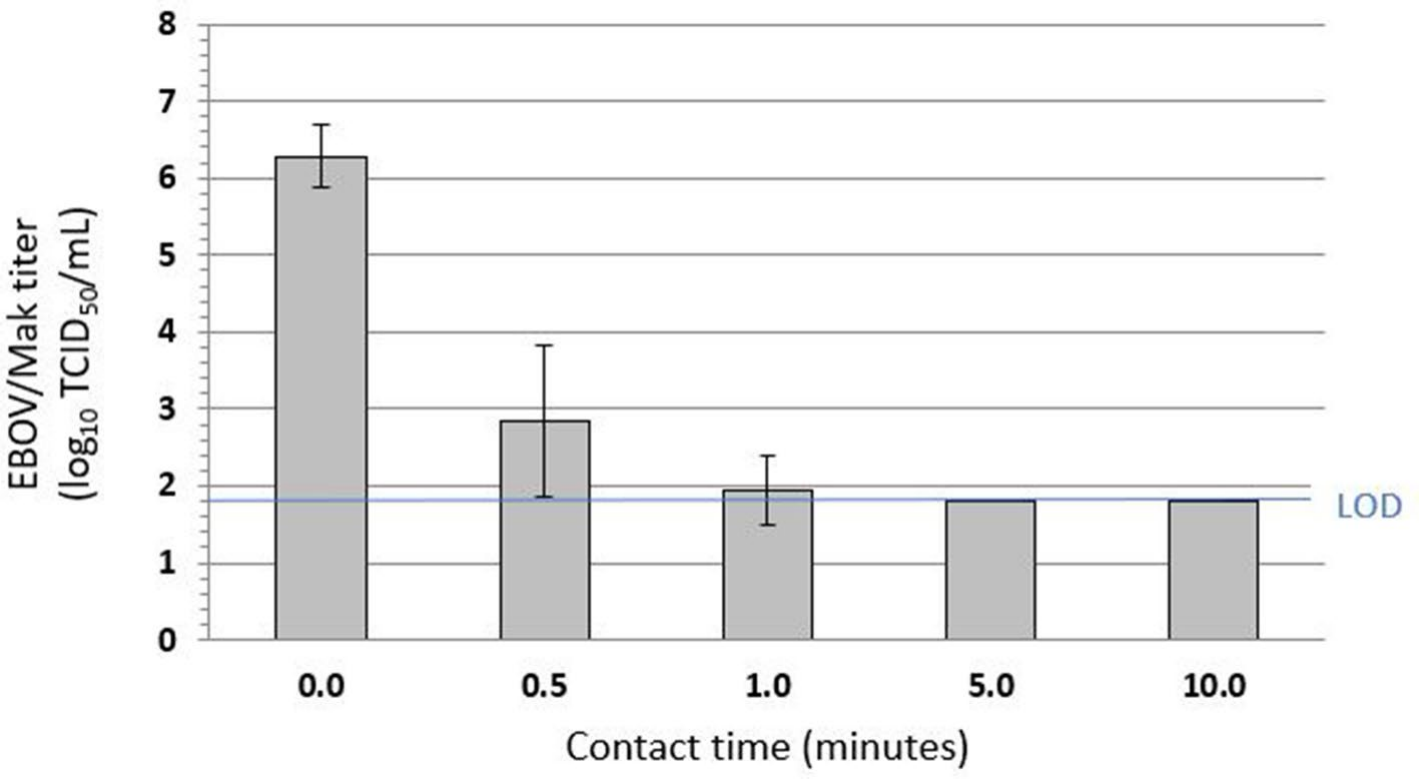
Time kinetics for EBOV/Mak virus inactivation by ethanol disinfectant spray (EDS) at ambient temperature. The values indicated represent the mean ± standard deviation log_10_ titer of the post-neutralization samples determined in Vero E6 cells (n = 3 technical replicates obtained for 3 lots of EDS). The input virus titer in tripartite soil load was found to be 6.3 ± 0.4 log_10_ TCID_50_/mL. The limit of detection (LOD) of the assay (1.8 log_10_ TCID_50_/mL) was determined by cytotoxic effects of the undiluted neutralized samples on Vero E6 cells.

The efficacy of a disinfectant spray containing 58% ethanol (EDS) for inactivating EBOV/Mak dried within an organic load on stainless steel carriers was evaluated per ASTM E2197-11^9^. Three lots of EDS were evaluated without dilution, with contact times of 0.5, 1, 5, and 10 min at ambient temperature (~ 21 °C). An initial virus load of ~ 6.3 log_10_ tissue culture infectious dose_50_ (TCID_50_) was dried on the carriers and then exposed to EDS. The infectious EBOV/Mak titer recovered from the carriers was reduced by > 5 log_10_ to the LOD of the assay within 5 min (Fig. 3).

The results of the plate safety test for EDS are shown in Table 2. Detectable EBOV/Mak was still present after contact times of 0.5 and 1 min but was complete (no virus detected) within 5 min of contact time on carriers (Fig. 5 and Table 3).

**Table 2:** Plate safety test results for inactivation of EBOV/Mak by ethanol disinfectant spray (EDS).

Test condition (contact time)	EDS		
	Lot 1	Lot 2	Lot 3
Negative control	−, −, −	−, −, −	−, −, −
N + EDS	−	−	−
EDS (0.5 min)	−, +, +	+, +, +	+, +, +
EDS (1 min)	−, −, −	−, −, −	−, +, −
EDS (5 min)	−, −, −	−, −, −	−, −, −
EDS (10 min)	−, −, −	−, −, −	−, −, −

The Vero E6 cultures were inoculated with undiluted neutralized samples and passaged twice. +, viral GFP observed; −, viral GFP not observed, or no cytotoxicity observed for N + EDS (neutralizer + EDS cytotoxicity control).

### Efficacy of inactivation of EBOV/Mak on carriers by 70% ethanol

**Figure 4:**
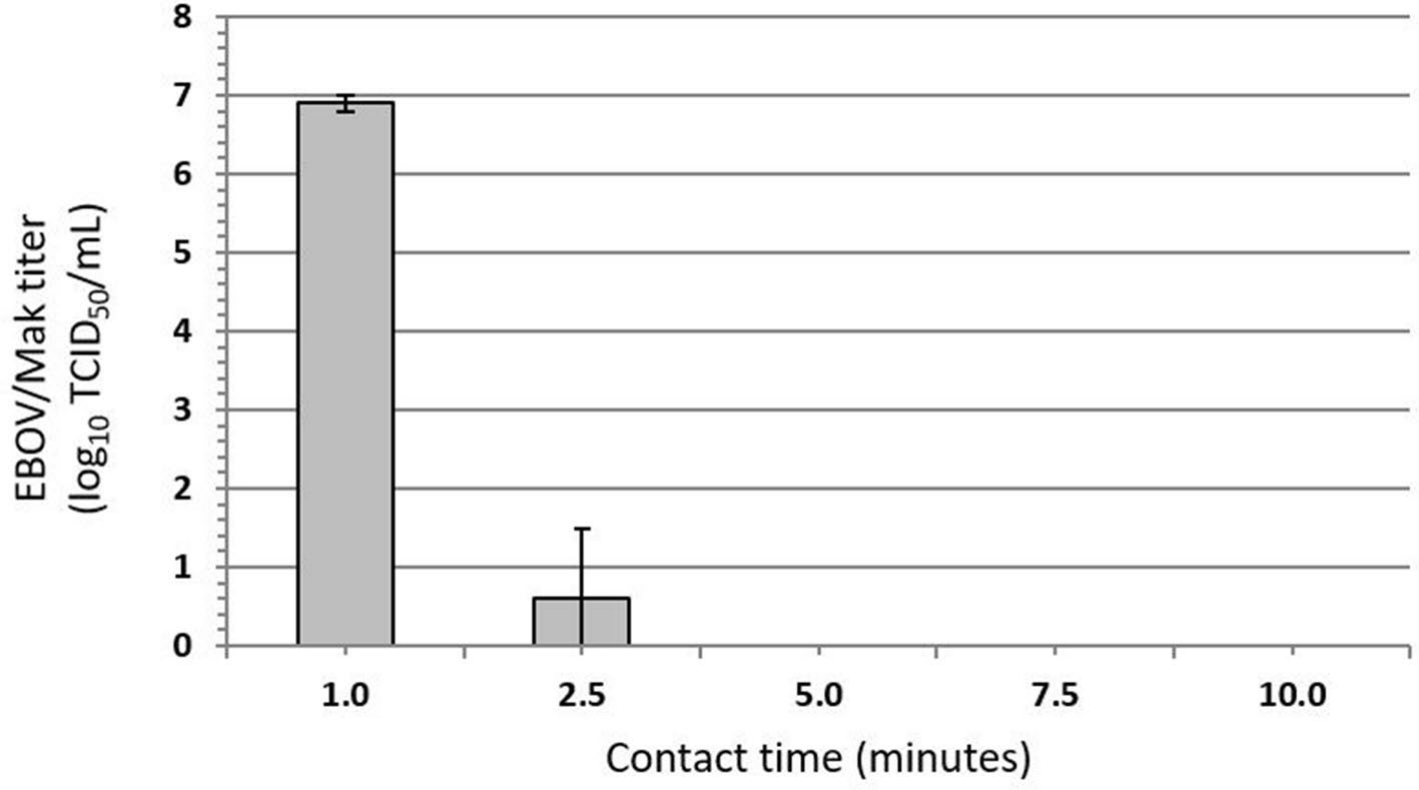
Time kinetics for EBOV/Mak virus inactivation by 70% ethanol at ambient temperature. The values indicated represent the mean ± standard deviation log_10_ titer of the post-neutralization samples determined in Vero E6 cells (n = 3 technical replicates obtained for 3 separate trials). No virus was detected at the 5-, 7.5-, and 10-min time points. The input virus titer in tripartite soil load was found to be 6.9 ± 0.3 log_10_ TCID_50_/mL (modified from Cook et al. [11]).

The efficacy of a 70% ethanol solution in hard water for inactivating EBOV/Mak dried within an organic load on stainless steel carriers was evaluated per ASTM E2197-11^9^. Contact times of 0, 1, 2.5, 5, 7.5, and 10 min were evaluated^11^ at ambient temperature (~ 27 °C). An initial virus load of ~ 6.8 log_10_ tissue culture infectious dose_50_ (TCID_50_) was dried on the carriers in the presence of the tripartite soil load^9^ and then exposed to the ethanol solution. Infectious EBOV/Mak titers recovered from the carriers were reduced by > 5 log_10_ within 2.5 min (Fig. 4). In this assay, the neutralized samples did not display cytotoxicity to the Vero E6 detector cells, so the limit of detection of the assay was essentially 1 TCID_50_/mL.

### Efficacy of inactivation of EBOV/Mak on carriers by sodium hypochlorite

**Figure 5:**
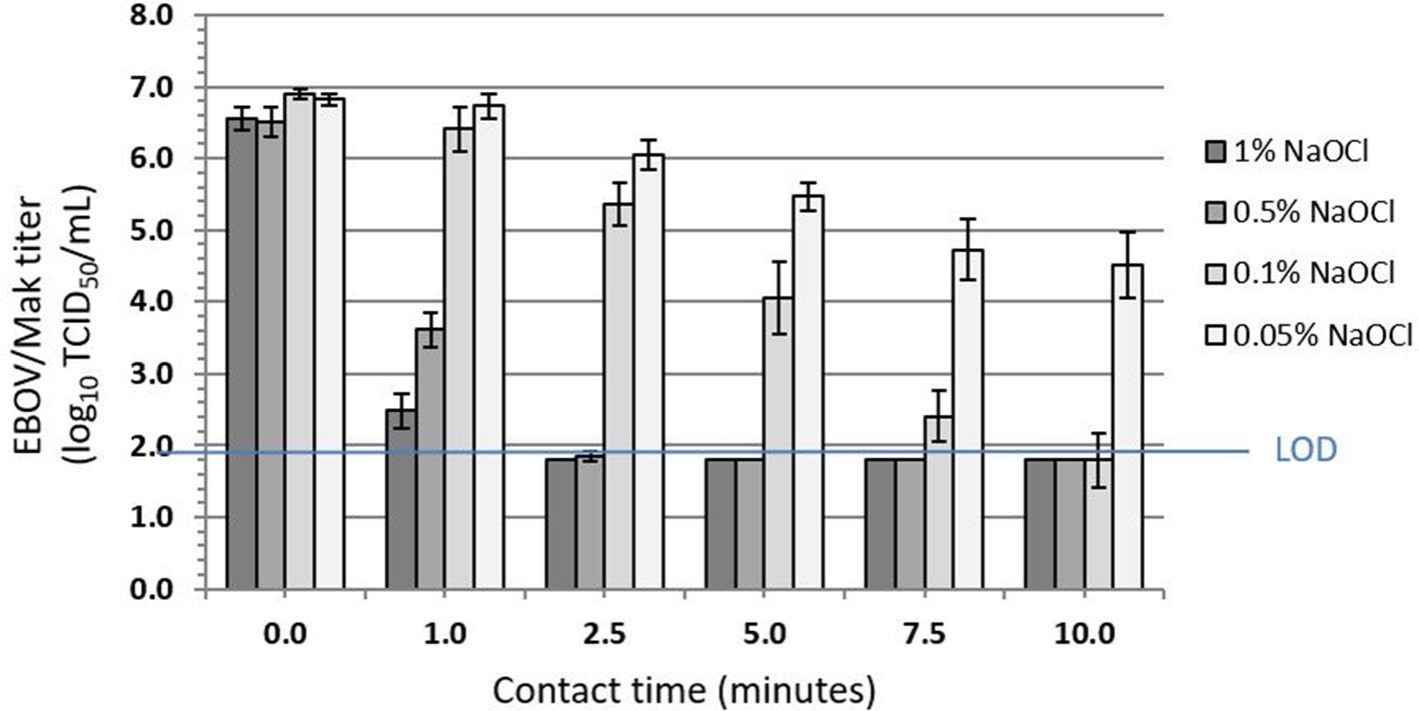
Time kinetics for EBOV/Mak virus inactivation by varying concentrations of sodium hypochlorite (NaOCl) at ambient temperature. The values indicated represent the mean ± standard deviation log_10_ titer of the post-neutralization samples determined in Vero E6 cells (n = 3 technical replicates obtained for 3 separate trials). The input virus titer in tripartite soil load was found to be 6.9 ± 0.3 log_10_ TCID_50_/mL. The limit of detection (LOD) of the assay (1.8 log_10_ TCID_50_/mL) was determined by cytotoxic effects of the undiluted neutralized samples on Vero E6 cells (modified from Cook et al.[11]).

The efficacy of NaOCl solutions for inactivating EBOV/Mak dried within an organic load on stainless steel carriers was evaluated^11^ per ASTM E2197-11^9^. Efficacy was evaluated^11^ at four NaOCl concentrations (0.05%, 0.1%, 0.5%, and 1%) in hard water, with contact times of 1, 2.5, 5, 7.5, and 10 min at ambient temperature (~ 27 °C). An initial virus load of ~ 6.8 log_10_ tissue culture infectious dose_50_ (TCID_50_) was dried on the carriers in the presence of the tripartite soil load^9^ and then exposed to NaOCl. At each concentration evaluated, the infectious EBOV/Mak titer recovered from the carriers was reduced by > 5 log_10_ to the LOD of the assay within five min (Fig. 5).

The results of the plate safety test conducted for NaOCl concentrations of 0.5% and 1% are shown in Table 3. Detectable infectious EBOV/Mak was still present after contact times of 1 and 2.5 min. Inactivation was complete (no virus detected) for these NaOCl concentrations within 5 min contact time on carriers (Fig. 5 and Table 3)

**Table 3:** Plate safety test results for inactivation of EBOV/Mak by the 0.5% and 1% NaOCl concentrations.

Test condition (contact time)	0.5% NaOCl			1% NaOCl		
	Trial 1	Trial 2	Trial 3	Trial 1	Trial 2	Trial 3
Negative control	−, −, −	−, −, −	−, −, −	−, −, −	−, −, −	−, −, −
N + NaOCl	−, −, −	−, −, −	−, −, −	−, −, −	−, −, −	−, −, −
Positive control	+, +, +	+, +, +	+, +, +	+, +, +	+, +, +	+, +, +
NaOCl (1 min)	+, +, +	+, +, +	+, +, +	+, +, +	+, +, +	+, +, +
NaOCl (2.5 min)	+, −, +	−, +, −	+, +, +	+, +, +	−, +, +	+, +, +
NaOCl (5 min)	−, −, −	−, −, −	−, −, −	−, −, −	−, −, −	−, −, −
NaOCl (7.5 min)	−, −, −	−, −, −	−, −, −	−, −, −	−, −, −	−, −, −
NaOCl (10 min)	−, −, −	−, −, −	−, −, −	−, −, −	−, −, −	−, −, −

The Vero E6 cultures were inoculated with undiluted neutralized samples and passaged twice. +, viral CPE observed; −, viral CPE not observed, or no cytotoxicity observed for N + NaOCl (neutralizer + NaOCl cytotoxicity control) (modified from Cook et al.^11^).

.

## Discussion

Filoviruses such as Ebola virus are capable of persisting for weeks on non-porous surfaces such as glass and plastic^12^. If high-touch environmental surfaces (HITES) are contaminated during outbreaks, these might serve as sources of infection of healthcare workers and other non-infected persons through the intermediacy of the hand. Targeted decontamination of such HITES therefore represents an opportunity for interrupting the spread of the virus.

The low minimum infectious dose of the Ebola virus in humans (estimated to be 1 to 10 infectious units)^13,14^ and the lethality (~ 41%) of the associated hemorrhagic disease^15^ mean that risk mitigation in the form of surface decontamination must be effective beyond the typical expectation of a 3–4 log_10_ reduction in viral titer. The US Environmental Protection Agency (EPA) stated in its 2012 disinfectant product guidance^16^ that “The product should demonstrate complete inactivation of the virus at all dilutions. If cytotoxicity is present, the virus control titer should be increased to demonstrate a ≥ 3 log_10_ reduction in viral titer beyond the cytotoxic level.” Most microbicides and/or the neutralizing agents used in determining inactivation kinetics are cytotoxic to the cells used in TCID_50_ assays used to determine efficacy. This impacts the sensitivity (limit of detection of the TCID_50_ assay). In the absence of cytotoxicity, a 4-log_10_ reduction in viral titer is considered to be effective. These requirements were recently modified in the 2018 revision of the EPA guidance^17^, with the new requirements being: In the revised guidance, a valid test requires (1) that ≥ 4.8 log_10_ of infectivity per carrier be recovered; (2) “the product should demonstrate a ≥ 3 log_10_ reduction on each surface in the presence or absence of cytotoxicity”; and (3) “if cytotoxicity is present, the virus control titer should be increased if necessary to demonstrate a ≥ 3 log_10_ reduction in viral titer on each surface beyond the cytotoxic level.” Note that in this revised guidance, an efficacious product does not need to demonstrate complete inactivation at all dilutions. That is, some residual infectious virus may be allowed.

In the case of the Ebola virus, we believe that the efficacy of a microbicide must be demonstrated by reduction of viral load to undetectable levels. When cytotoxicity to detector cells occurs in the TCID_50_ assay, an alternative means must be used to demonstrate complete inactivation (i.e., elimination of any residual infectious virus). In our experiments, this has been accomplished through the method referred to as the “plate safety assay”. This abrogates the issue of cytotoxicity through inoculation of undiluted neutralized test samples into 6-well cultures of Vero E6 cells, and then by conducting serial passages of the inoculated cells. A negative result in this method provides evidence of complete inactivation of any infectious virus.

Here, we present our data on the virucidal efficacy of varying concentrations of PCMX and a single concentration of a formulated ethanol spray against the Makona strain of Ebola virus (EBOV/Mak), together with previously published data^11^ on the efficacy of 70% ethanol and varying concentrations of sodium hypochlorite against EBOV/Mak. Each of these data sets were obtained from the same testing facility using the same methodology. As a result, the virucidal efficacy results should be directly comparable. The carrier inactivation data for EBOV/Mak presented here demonstrate that a variety of microbicides should be useful for effective inactivation of Ebola virus on stainless steel surfaces. These microbicides include 70% ethanol at contact times ≥ 5 min, NaOCl at concentrations of 0.5% or greater, at contact times ≥ 5 min, PCMX at concentrations of 0.48% and contact time of ≥ 5 min, and the EDS used as supplied at contact time ≥ 5 min. Under these conditions, no residual EBOV/Mak virus was detectable (≥ 6.3 log_10_ inactivation) as indicated by the TCID_50_ assay and the plate safety assay.

Cook et al.^11^ previously examined the efficacy of 70% ethanol and several concentrations of sodium hypochlorite against three variants of the Ebola virus (Mayinga, Kikwit, and Makona). The authors found that the Makona variant was somewhat less susceptible to the lower concentrations of sodium hypochlorite, while being similarly susceptible to 70% ethanol and higher concentrations of sodium hypochlorite^11^. We therefore expect that the efficacy data obtained here for the Makona variant should apply also to other outbreak variants of Ebola virus.

Sodium hypochlorite 0.5% solutions are recommended by the CDC^18^ as an example of a suitable disinfectant solution for hemorrhagic fever viruses. Smither et al.^19^ use a similar experimental design (i.e., a combination of TCID_50_ assay and passage of neutralized solutions in flasks to rule out residual infectious virus) to study the disinfection of Ebola virus Yambuku-Ecran (EBOV-Ecran) on aluminum carriers by 0.75% NaOCl in tap water for 10 min contact time. In this case, complete inactivation of the 1.5 × 10^5^ TCID_50_/mL dried on the carrier was achieved^19^. No organic matrix was used in the studies for EBOV/Ecran. Additional studies by Smither et al.^20^ evaluated disinfection of EBOV/Mak dried in a blood matrix on stainless steel or aluminum surfaces. In this study, 0.5 and 1% NaOCl inactivated EBOV/Mak dried in tissue culture medium or deposited in wet blood to the LOD of the assay (10 TCID_50_/mL) in 15 min. This suggested that inactivation of EBOV in the presence of dried blood was particularly challenging for NaOCl, more challenging that is, than virus dried in tissue culture medium. Only a 5-min contact time with 5% peracetic acid solutions was capable of completely inactivating the EBOV/Mak dried in blood in that study^20^.

These results from Cook et al.^11^ using 70% ethanol and NaOCl solutions and from the present study using PCMX solutions and EDS demonstrated ≥ 6 log_10_ inactivation of EBOV/Mak dried on steel by EDS or solutions of NaOCl (≥ 0.5%), PCMX (≥ 0.12%), or 70% ethanol at contact times ≥ 5 min. Higher log inactivation values might actually have been possible, but were not able to be determined in the present study due to limitations in the amount of challenge virus that could be applied. These four disinfectants display sufficient inactivation efficacy for Ebola virus at reasonably short contact times that may be practically achieved in the field. Use of these disinfectants for surface decontamination in the field or in healthcare settings therefore has the potential to reduce infectious Ebola virus load on those surfaces and to reduce spread of virus from infected to non-infected individuals.

## Methods

Methods for the previously reported Ebola variant carrier inactivation studies involving ethanol and sodium hypochlorite were described in Cook et al.^11^ and have not been reproduced here.

### Cell line, virus, and medium

African green monkey Vero E6 cells (ATCC CRL-1586; American Type Culture Collection, Manassas, VA, USA) were maintained at 37 °C/5% CO_2_ in Dulbecco’s modified Eagle medium (DMEM; HyClone, Logan, UT, USA) supplemented with 10% fetal calf serum (FCS; Gibco, Grand Island, NY, USA) and 10 units/mL penicillin/streptomycin (Gibco). Ebola virus Makona variant (EBOV/Mak; Ebola virus/H. sapiens-tc/GIN/2014/Makona-C05; GenBank Accession No. KJ660348) was obtained from a clinical isolate. For the studies on PCMX and EDS, the isolate was biotechnologically engineered to express green fluorescent protein (GFP).

### Stock virus preparation

A characterized stock of EBOV/Mak virus was prepared by infecting five T-175 flasks of Vero E6 cells expressing green fluorescent protein (eGFP) at ~ 80% confluency at a multiplicity of infection (MOI) of 0.01. The eGFP was evident by day 3 post-infection. At ~ 9 days post-infection, marked cytopathic effects (CPE) were also observed, at which time the flasks were frozen at − 70 °C. The conditioned medium from thawed flasks was clarified by low-speed centrifugation (4,500×*g*) for 10 min. The supernatants were pooled and overlaid onto 20% w/v sucrose cushions prepared in Tris-NaCl-EDTA buffer. After centrifugation at 133,907×*g* for 2 h, the resulting viral pellets were resuspended in virus culture medium (VCM; DMEM containing 2% FCS and 10 units/mL penicillin/streptomycin) overnight at 4 °C. The resuspended virus was pooled and aliquoted into usable amounts and frozen at − 70 °C until needed. Stock virus titers were determined to be > 8.4 log_10_/mL by TCID_50_ assay, with titer calculation following the Reed-Muench method^21^. All EBOV/Mak manipulations were carried out in a BSL-4 laboratory at the Canadian Science Centre for Human and Animal Health, Winnipeg, Manitoba, Canada.

### Microbicide solution preparation

Three concentrations (0.12%, 0.24%, and 0.48%) of three lots of PCMX (lot# 812314, 812315, and 705212) were prepared from the ready-to-use commercial product by dilution in 440 ppm hard water^9^ (prepared as 1 L deionized water supplemented with 0.4 g calcium carbonate) on the day of assay performance. The resulting solutions were inverted to mix and were used within 4 h of preparation. The EDS formulations containing 58% ethanol were used without further dilution. Three independent lots of EDS (lot# 2231-1, 1858-64, and 2233-7) were evaluated.

### Neutralization

Methods for evaluating neutralizing agents are described in the Supplemental Materials section.

### Efficacy testing on carriers

Inactivation efficacy testing for microbicides was performed in carrier studies (Fig. 1) conducted at ambient temperature (21 °C) per ASTM E2197-11^9^. Inocula for stainless steel carriers were prepared fresh on the day of assay by mixing previously frozen concentrated EBOV/Mak eGFP virus with a tripartite soil load^9,10^. The virus in tripartite soil load (10 µL) was added to sterile scored stainless steel test carriers and air dried at ambient temperature for 1 h. After drying, carriers were placed in 6-well plates and 50 µL of microbicide was added to each carrier and incubated for 30 s, 1 min, 5 min, and 10 min contact times. Input virus titers determined for the various virucidal efficacy experiments may have varied as a result of differences in the titers of the virus stocks used in the individual studies. Such differences had no impact on the validity of the studies. Per the standard followed, ASTM E2917-11, at least 4 log_10_ reduction of virus should be exhibited to demonstrate efficacy, and the input titers used were high enough to allow demonstration of this log reduction after accounting for possible toxicity. A positive control (50 µL of VCM in lieu of microbicide) was used for each assay performed. At the end of each exposure time point, the microbicide was neutralized by adding 950 µL of appropriate neutralizing agent to the test carriers and to the positive controls last. The carriers were rinsed via pipetting 20 times with neutralized solution to elute any dried virus from the carriers into the solution. A 500-µL portion of the positive control and each neutralized test solution was ten-fold serially diluted in VCM, and 50 µL of the resulting dilutions were added to 96-well plates containing Vero E6 cells (n = 5 replicates per dilution). After a 45-min adsorption period, 150 µL of VCM were added to each well. The Vero E6 cell wells were scored 9–14 days post-infection for green fluorescence and cytopathic effect (CPE) and virus titers (TCID_50_) were calculated according to the Reed-Muench method^21^. Similar titers for the Virus Positive Control condition were obtained regardless of the readout used (GFP vs. CPE) (see Supplemental Materials). The log_10_ reduction values achieved by the various exposure time points were calculated by subtracting the post-disinfection log_10_ TCID_50_ values from the log_10_ titers obtained for the corresponding positive controls. The undiluted (10^0^) neutralized PCMX and EDS solutions displayed toxic effects in the Vero E6 cells and could not be evaluated for viral titer in the TCID_50_ assay. A plate safety test was employed to evaluate surviving EBOV/Mak virus in these samples. Triplicate 500-µL aliquots of each undiluted neutralized test sample were added immediately following neutralization to 7 mL of VCM in a six-well plate of Vero E6 cells at ~ 80% confluency. The cultures were incubated 9–14 days and scored for presence of green fluorescence and CPE.

## Supplementary information

Supplementary file1
