# Supplementary material for: Efficacy of microbicides for inactivation of Ebola–Makona virus on a non-porous surface: a targeted hygiene intervention for reducing virus spread
Source: Sci Rep. 2020 Sep 17;10:15247. doi: 10.1038/s41598-020-71736-x (PMC7498580; doi:10.1038/s41598-020-71736-x)

**Supplemental Material for**

**Efficacy of Microbicides for Inactivation of Ebola-Makona Virus on a Non-Porous Surface – A Targeted Hygiene Intervention for Reducing Virus Spread**

**Todd A. Cutts, Catherine Robertson, Steven S. Theriault, Raymond W. Nims**, **Samantha B. Kasloff, ,Joseph R. Rubino, and M. Khalid Ijaz**

**Measurement of Viral Titer using Viral Cytopathic Effect vs. Green Fluorescent Protein**

Green fluorescent protein (GFP) in Vero E6 cells inoculated with EBOV/Mak could be detected earlier than viral cytopathic effect (CPE). This is because even a few cells expressing GFP could be visualized microscopically, while CPE requires longer to develop. The titer of the EBOV/Mak Virus Positive Control was calculated on varying days post-inoculation of Vero E6 cells using both GFP and CPE as readouts. The results, shown in Supplemental Figure S1, demonstrate that the eventual titer obtained for the Virus Positive Control using the two readouts was approximately equivalent.

**Supplemental Figure S1.** Plot of EBOV/Mak Virus Positive Control determination based on viral cytopathic effect (CPE) vs. green fluorescent protein (GFP) in Vero E6 cells based on scoring on days 0 through 14 post-inoculation.


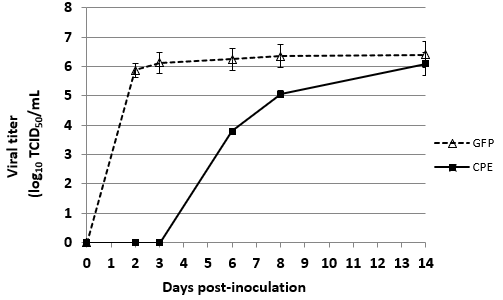


**Microbicide Neutralization Effectiveness Assay Method**

Prior to preforming the efficacy assay of PCMX and EDS (ethanol disinfectant spray) on virus-contaminated surfaces, a neutralization assay was required to determine an effective neutralizing agent against the formulations being tested. Specifically, this assay evaluated the interaction of neutralizers and disinfectants with the host cell line and the virus to ensure that the effects observed were due to the infectious virus and were not a byproduct of chemical toxicity from the test reagents involved. Combinations of neutralizers, disinfectants, and test virus were utilized to account for this.

One day prior to testing, 96-well microtiter plates of Vero E6 cells were seeded to obtain approximately 80% monolayer confluency on the day of the neutralization assay. This ensured cells were in logarithmic growth phase, ideal for infectivity studies, and would not overgrow before the final scoring on day 14 post-inoculation.

On the day of neutralization assay, candidate neutralizing reagents (DMEM+10% fetal calf serum [FCS]+10 units/mL penicillin/streptomycin, 1× Letheen broth alone, and 1× Letheen Broth + virus culture medium [VCM; DMEM containing 2% FCS and 10 units/mL penicillin/streptomycin]) were prepared fresh. In addition, a low titer virus stock (10^5^ to 10^6^ TCID_50_/mL) was also prepared with only 10µl inoculum used for the neutralization control. All neutralization controls were performed in replicates of three over a single experiment. Neutralizer controls included the following:

**Negative Control:** Cells were cultured in VCM and used as the basis for comparison for evaluating cytotoxicity, viral cytopathic effect (CPE), or green fluorescent protein (GFP).

**Neutralizer Control (cells):** This was used to determine the effect of the neutralizer used on the overall health of the Vero E6 cells used in the assay. The neutralizer to be evaluated was ten-fold serially diluted in VCM and 50 µL were added to Vero E6 cells in replicates of 5 for each dilution from 10^0^ (neat) to 10^-3^. Cells were scored for cytotoxicity 14 days post-inoculation.

**Neutralizer Disinfectant Control (cells):** This was used to determine the Limit of Detection of the cell-based infectivity assay, as the combination of disinfectant and neutralizer might adversely impact the ability of the cells to detect viable virus. A 50-µL quantity of microbicide formulation was added to 950 µL of neutralizer. Neutralizer-disinfectant control was 10-fold serially diluted in VCM with 50 µL added to Vero E6 cells in replicates of 5 for each dilution from 10^0^ (neat) to 10^-3^. Three technical replicates were used for one experiment. Cells were scored for cytotoxicity 14 days post-inoculation.

**Virus Positive Control:** This was used to determine the virus titer exposed to disinfectant**.** The positive virus control was performed by adding 10 µL of diluted low-titer virus (10^2^ to 10^3^ TCID_50_/10 µL) in tripartite soil load was added to 990 µL of VCM. Virus control was 10-fold serially diluted in VCM and 50 µl added to Vero E6 cells in replicates of 5 for each dilution from 10^0^ (neat) to 10^-3^. Cells were scored for CPE or GFP 14 days 14 days post-inoculation.

**Neutralizer + Virus Control:** To account for the effect of the neutralizer acting on the virus, 10 µL of low-titer virus (10^2^ to 10^3^ TCID_50_/10 µL) in tripartite soil load was added to 990 µL of neutralizer. Neutralizer + Virus Control was 10-fold serially diluted in VCM and 50 µL added to Vero E6 cells in replicates of 5 for each dilution from 10^0^ (neat) to 10^-3^. Cells were scored for CPE or GFP 14 days post-inoculation. Results were compared to the virus positive control.

**Neutralizer + Disinfectant + Virus Control:** To account for the ability for the neutralizer to affectively mitigate (neutralize) the effects of the microbicide tested, a 50-µL quantity of test microbicide was added to 940 µL of neutralizer and mixed. Ten µL of low-titer virus (10^2^ to 10^3^ TCID_50_/10 µL) were added and the mixture was incubated for 10 minutes at ambient temperature. The resulting control was 10-fold serially diluted in VCM and 50 µL were added to Vero E6 cells in replicates of five for each dilution from 10^0^ (neat) to 10^-3^. Cells were scored for CPE or GFP 14 days post-inoculation. Results were compared to the virus positive control.

**Results for Neutralization Effectiveness Testing**.

**Neutralization of para‐chloro‐meta‐xylenol** (**PCMX).** Neutralization of the microbicidal effects of PCMX (0.12%, 0.24%, and 0.48% in hard water) was achieved with dilution of the microbicide/virus mixture in 1 mL of Letheen Broth alone (Supplemental Figure S2).

**Supplemental Figure S2.** Ability of Letheen broth to neutralize the EBOV/Mak-inactivating effects of 0.12%, 0.24%, or 0.48% PCMX. TCID_50_/mL, tissue culture infectious dose_50_/mL. No significant differences were observed by unpaired *t*-test (*P* < 0.05) between Virus Positive Control, Neutralizer + Virus Control, and Neutralizer + Disinfectant + Virus Control conditions.


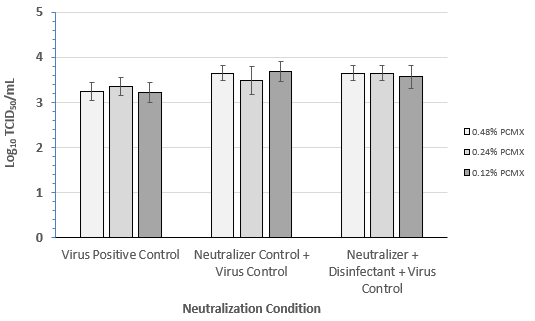


**Neutralization of Ethanol Disinfectant Spray (EDS).** Neutralization of the microbicidal effects of EDS was achieved with dilution of the microbicide/virus mixture in 1 mL of DMEM + 10% FCS +10 units/mL penicillin/streptomycin (Supplemental Figure S3).

**Supplemental Figure S3.** Ability of DMEM+10% FCS+10 units/mL penicillin/streptomycin to neutralize the EBOV/Mak-inactivating effects of EDS. Abbreviations used: TCID_50_/mL, tissue culture infectious dose_50_/mL. No significant differences were observed by unpaired *t*-test (*P* < 0.05) between Virus Positive Control, Neutralizer + Virus Control, and Neutralizer + Disinfectant + Virus Control conditions.


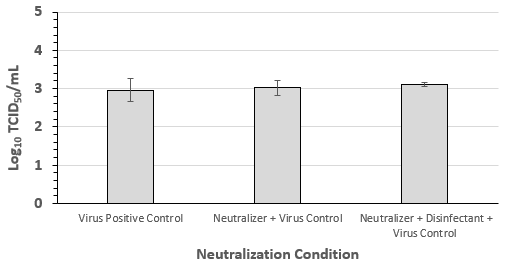

Supplement: Supplementary file 1 — Supplementary file1 [file 41598_2020_71736_MOESM1_ESM.docx]
